# LncRNA SNHG1 promotes tumor progression and cisplatin resistance through epigenetically silencing miR-381 in breast cancer

**DOI:** 10.1080/21655979.2021.1996305

**Published:** 2021-11-22

**Authors:** Mingkun Zhang, Liu Yang, Lan Hou, Xueyuan Tang

**Affiliations:** a Department of Thyroid, Breast and Vascular Surgery, Xijing Hospital, the Fourth Military Medical University, Xi’an, China; b Department of Reproductive Endocrinology, Xi’an International Medical Center Hospital, Xi’an, China

**Keywords:** Breast cancer, long non-coding RNA, small nucleolar RNA host gene 1, enhancer of zeste homolog 2, miR-381

## Abstract

The long-non-coding RNA (lncRNA) small nucleolar RNA host gene 1 (SNHG1) is a known cause of tumorigenesis. Nevertheless, it’s yet unclear how lncRNA SNHG1 influences breast cancer. Herein, we explored the mechanisms through which SNHG1 modulates breast cancer tumor progression. Our findings demonstrated that SNHG1 is significantly upregulated in breast cancer tissues and cells. High SNHG1 levels were closely linked to reduced survival rates in breast cancer patients. SNHG1 silencing has been shown to inhibit the proliferative, migratory, and invasive activity of breast cancer cells. Moreover, SNHG1 silencing enhanced cisplatin (DDP) sensitivity of these cells through improving DDP-induced cell apoptosis. Mechanistically, SNHG1 was found to interact with enhancer of zeste homolog 2 (EZH2), recruiting EZH2 to trigger trimethylation of histone H3 lysine 27 (H3K27me3), thus epigenetically inhibiting miR-381 transcription in these cells. Overexpression of miR-381 inhibited tumor progression and sensitized cells to the chemotherapeutic reagent DDP. More importantly, rescue experiments demonstrated that miR-381 inhibition could inverse the tumor-suppressive effect of SNHG1 silencing in breast cancer. In summary, SNHG1 silencing suppressed tumor progression and overcame breast cancer cell DDP resistance via the epigenetic suppression of miR-381 expression. Our study revealed that SNHG1 served as a novel therapeutic target for breast cancer chemoresistance.

## 1. Introduction

Breast cancer is one of the most prevalent gynecologic malignant tumors and represents a major threat to the health of females [1]. Although great advances have been made in breast cancer therapeutics, the prognosis of patients remains at an unsatisfactory level [2]. Surgical tumor removal and adjuvant chemotherapy remain the primary treatments for breast cancer. Nevertheless, as these tumors are very aggressive and often chemoresistant, patient’s treatment remains challenging [3,4]. Therefore, to sequentially elucidate the mechanism is imperative for breast cancer treatment, as novel target identification has the potential to guide drug development and appropriate treatment.

LncRNAs are longer than 200 nucleotides and lack the ability to code protein [5]. They are important in terms of their extensive regulatory actions in the development of human cancers [6–8]. Additionally, lncRNAs are vital regulators of chemoresistance in malignancies [9–11]. SNHG1 is a key lncRNA that is expressed in prostate cells that has been shown to promote the development of prostate cancer [12]. Moreover, up-regulated SNHG1 could exert an oncogenic role in osteosarcoma, contributing to tumor progression and poor prognosis in osteosarcoma patients [13]. Recently, SNHG1 was revealed to be upregulated and promoted tumor progression in breast cancer [14]. However, whether SNHG1 affects breast cancer chemoresistance remains uncertain, making this a key topic worthy of further experimental evaluation.

miRNAs are non-coding RNAs with 21–24 nucleotides, which bind to the 3ʹ untranslated regions (3ʹUTR) of target mRNAs to promote their degradation, thereby reducing gene expression [15,16]. miRNAs are involved into tumorigenesis and chemoresistance [17]. miR-381 was reported to be down-regulated and act to suppress tumor progression in breast cancer through targeting CXC motif chemokine receptor type 4 (CXCR4) [18]. Furthermore, miR-381 can target Multidrug Resistance 1 (MDR1) to suppress breast cancer DDP resistance [19]. The control of miR-381 in breast cancer, however, has yet to be characterized.

It has been suggested that SNHG1 suppresses host cell gene expression through its ability to target the polycomb repressive complex 2 (PRC2) subunit enhancer of zeste homolog 2 (EZH2) [20,21]. Chipbase analyses suggested that EZH2 was capable of binding miR-381, which could attribute to DNA hypermethylation [22]. Together with the findings of the previous studies, we suspected that SNHG1 may epigenetically suppress miR-381 expression via recruiting EZH2 in breast cancer cells. Therefore, the aim of our study was to reveal the function and epigenetic mechanism of lncRNA SNHG1 in breast cancer, which may provide for novel insights into therapeutic targets and functional mechanism for breast cancer.

## 2. Methods

### 2.1. Tissue and cell samples

Surgical specimens from human Luminal A, Luminal B, Basal and HER2-positive breast cancer tissues were acquired from 48 breast cancer patients, who had not received preoperative radiotherapy or chemotherapy, treated within Xijing Hospital, The Fourth Military Medical University. All patients provided consent and the study was approved by our ethics board. Breast cancer cell lines (MCF-7 & MDA-MB-231) and normal epithelial cell line (MCF-10A) were purchased from the ATCC and cultured as previously described [23].

### 2.2. Transfection

Empty pcDNA3.1, pcDNA-SNHG1, and siRNAs targeting SNHG1 (si-SNHG1#1, si-SNHG1#2, si-SNHG1#3) or EZH2 (si-EZH2) were purchased were synthesized by GenePharma (Shanghai, China). All transfections were performed with Lipofectamine 2000 (Invitrogen).

### 2.3. qRT-PCR

RNA was extracted from breast cancer tissues and MCF-7 and MDA-MB-231cells using TRIzol and used to generate cDNA with M-MLV Reverse Transcriptase (Invitrogen). Next, an ABI Prism 7900 instrument was used to perform qRT-PCR using SYBR green (VeriQuest Fast, Thermo Fisher). Relative expression was analyzed using 2^−∆∆Ct^ method with U6 and GAPDH as internal reference. Primer sequences are shown in Table 1.

**Table 1.** List of primer sequences used for qRT‑PCR

miRNA/gene name	Sequences
SNHG1‑forward	5ʹ-AGGCTGAAGTTACAGGTC-3’
SNHG1‑reverse	5ʹ-TTGGCTCCCAGTGTCTTA-3’
miR‑381‑forward	5ʹ-TACTTAAAGCGAGGTTGCCCTT-3’
miR‑381‑reverse	5ʹ-GGCAAGCTCTCTGTGAGTA-3’
GAPDH‑forward	5ʹ-AAGGTGAAGGTCGGAGTCA-3’
GAPDH‑reverse	5ʹ-GGAAGATGGTGATGGGATTT-3’
U6‑forward	5ʹ-CTCGCTTCGGCAGCACA-3’
U6‑reverse	5ʹ-TGGTGTCGTGGAGTCG-3’

### 2.4. Cell proliferation and DDP sensitivity assays

MTT assays were used to assess cell proliferation rates. The viability of cells treated with different concentrations of DDP (0.1, 1, 5, 10, 25, 50, 100 μM) was determined using MTT assays. DDP sensitivity was determined using the IC50 value (half maximal inhibitory concentration) which was calculated using GraphPad Prism 7.0 Software.

### 2.5. Transwell assays

Transfected cells were added to the upper chambers of Matrigel coated wells (Corning, NY, USA) for invasion and migration assays, respectively. Cells in the upper chambers were cultured in media with 10% fetal bovine serum, whilst cells in the lower chambers were assessed for migration or invasion. After 24 h, noninvasive or non-migrating cells were carefully removed using a swab, and invaded or migrated cells were fixed with formaldehyde and stained with crystal violet. These cells were imaged and enumerated via light microscopy.

### 2.6. Apoptotic assessments

For the assessment of drug induced cell death, transfected cells treat with 2 μM DDP were treated for 48 h and stained with commercial PI and Annexin V staining kits. Cell apoptosis were assessed by flow cytometry [24].

### 2.7. Caspase activity assay

DDP treated cells were assessed for caspase activity using commercially available colorimetric kits (R&D Systems Inc., MN, USA). Absorbances were read at 405 nm on a microplate reader.

### 2.8. Subcellular location assay

MCF-7 cells were harvested in NP-40 lysis buffer on ice for 10 min and centrifuged at 4°C for 5 min. Cytoplasmic and nuclear fractions were isolated from the supernatants and pellets respectively, and qRT-PCR analysis was performed.

### 2.9. RNA immunoprecipitation (RIP)

Magna RIP Kits (Millipore) were used for interactions analyses. Briefly, cells were lysed in RIPA buffer and probed with anti-EZH2 (Millipore) or control IgG (Millipore)-antibodies conjugated to magnetic beads as previously described [25]. After incubation with proteinase K, the immunoprecipitated RNA was prepared for further qRT-PCR.

### 2.10. ChIP assays

The promotor sequence for miR-381 were obtained from UCSC (https://genome.ucsc.edu/). EZ Chromatin immunoprecipitation (ChIP) Kits (Millipore) were employed using control IgG (Millipore), anti-EZH2 (Abcam, MA, USA) or anti-H3K27me3 antibodies (Millipore). Precipitated DNA was subject to qRT-PCR analysis.

### 2.11. Luciferase assays

Reporter plasmids of miR-381 promoter were individually synthesized by GenePharma and co-transfected with pcDNA-SNHG1 or si-SNHG1 in MCF-7 cells for 48 h. Dual Luciferase Reporter Assays were performed using commercial kits (Promega).

### 2.12. Animal experiments

All animal studies described herein were approved by our institutional animal ethics committee. Briefly, we implanted the mice with E2 pellets before MCF-7 cell injection for tumor formation. Then, MCF-7 cells that had been transformed to stably expressing sh-SNHG1 or sh-con were implanted into the axils of six female BALB/c nude mice (six-week-old, n = 3 per group) from Slac Laboratory (Shanghai, China). After one-week, tumor volumes were measured and calculated every 7 days using the following equation (v = 0.5 × length × width^2^). Mice were sacrificed after 35 days and xenograft tumors were weighed. Tumors were collected and analyzed by qRT-PCR.

### 2.13. Data analysis

Data are shown as the mean ± standard error. Data were compared via student’s t-test and one-way ANOVAs using SPSS v16.0. *P* < 0.05 was the significance threshold.

## 3. Results

In our study, we predicted that the lncRNA SNHG1 may play a key role in breast cancer by epigenetically silencing miR-381. Therefore, we aimed to investigate the functional role of SNHG1/miR-381 axis in breast cancer. Our data revealed that SNHG1 was upregulated in breast cancer, and silencing SNHG1 suppressed tumor progression and cisplatin resistance of breast cancer cells. Moreover, miR-381 could be epigenetically suppressed by SNHG1 and miR-381 inhibition could partly reverse the effect of SNHG1 knockdown on breast cancer cells. In summary, our findings demonstrated that SNHG1 contributed to tumor progression and cisplatin resistance in breast cancer cells through epigenetically silence miR-381 via recruiting EZH2 on the promotor of miR-381, which may provide a novel mechanism and therapeutic target for breast cancer chemotherapy.

### 3.1. SNHG1 was overexpressed in breast cancer

**Figure 1.**
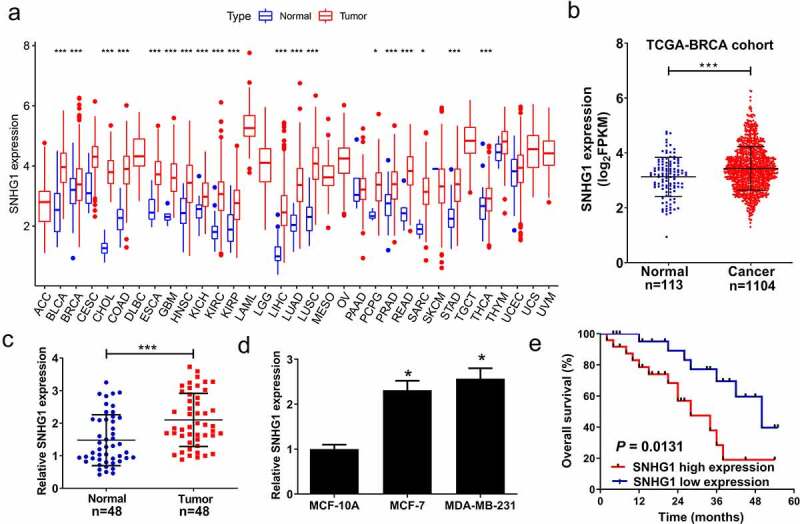
SNHG1 was upregulated in breast cancer tissues and cells. SNHG1 expression was analyzed in pan-Cancer datasets (a) and TCGA-BRCA dataset (b). SNHG1 expression was detected using qRT-PCR analysis in paired breast cancer tumor (n = 48) or adjacent normal (n = 48) tissues (c), and breast cancer cells (MCF-7 and MDA-MB-231) and human normal cells (MCF-10A) (d). (e) Kaplan-Meier curve was performed to evaluate the overall survival between low and high SNHG1 expression groups. *P < 0.05, ***P < 0.001

We first analyzed the expression of SNHG1 in pan-Cancer and TCGA-BRCA datasets. Our results confirmed that SNHG1 was up-regulated in pan-Cancer (Figure 1(a)) and TCGA-BRCA (Figure 1(b)) datasets. To further explore the role of SNHG1 in breast cancer development, we examined SNHG1 expression in tumor and adjacent normal tissues through qRT-PCR analysis. We found that SNHG1 expression was upregulated in tumor tissues (Figure 1(c)). Moreover, SNHG1 was significantly upregulated in breast cancer cells (Figure 1(d)), and correlated with shorter overall survival in breast patients (*P* = 0.0137) (Figure 1(e)). Taken together, upregulated SNHG1 is potentially associated with tumorigenesis in breast cancer.

### 3.2. SNHG1 knockdown suppressed breast cancer cell proliferative, migratory, and invasive activity

**Figure 2.**
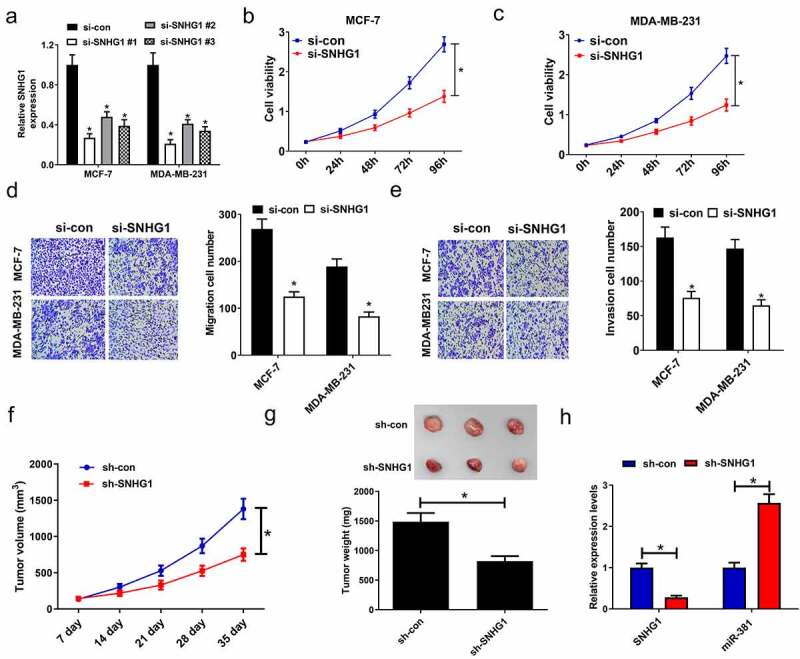
SNHG1 silencing inhibited cell proliferation, and migration and invasion in breast cancer cells. (a) qRT-PCR analysis of SNHG1 expression in MCF-7 and MDA-MB-231 cells transfected with siRNAs (si-SNHG1#1, si-SNHG1#2, si-SNHG1#3) or si-con. (b and c) MTT assay determined the cell viability of si-SNHG1- or si-con-transfected cells. (d) Migration assay in si- SNHG1- or si-con-transfected cells was performed. (e) Invasion assay was carried out to detect the invasion number of si-SNHG1- or si-con-transfected cells. (f) Tumor volume was monitored every 7 days after inoculation. MCF-7 cells stably expressing sh-SNHG1 or sh-con were inoculated into nude mice. (g) The tumors were excised at 35th days after inoculation and weighed. (h) qRT-PCR analysis of SNHG1 and miR-381 expression in xenograft tumors. *P < 0.05

To determine how SNHG1 expression influences breast cancer tumorigenesis, breast cancer cells were transfected with a panel of siRNAs targeting SNHG1, and verified the reduction of its expression, especially in the si-SNHG1 #1 group (Figure 2(a)). Therefore, si-SNHG1#1(named si-SNHG1 in further experiments) was selected to knockdown SNHG1 in the following studies. MTT assay revealed SNHG1 knockdown led to an inhibition of cell viability (Figures 2(b,c)). Meanwhile, silencing of SNHG1 resulted in decreased migration and invasion (Figures 2(d,e)). Given the role of SNHG1 *in vitro*, we investigated if SNHG1 knockdown suppressed tumor growth *in vivo*. MCF-7 cells infected with sh-SNHG1 or sh-con were inoculated into nude mice. We found that SNHG1 knockdown caused a noticeable inhibition of tumor growth in the sh-SNHG1 group relative to the sh-con group (Figures 2(f,g)). Furthermore, compared with the tumors in control group, an evident decline of SNHG1 expression and a striking elevation of miR-381 expression were found in the tumors after sh-SNHG1 knockdown, as determined by qRT-PCR (Figure 2(h)). These findings emphasized that SNHG1 silencing dramatically suppressed tumor growth, highlighting its importance in breast cancer progression and development.

### 3.3. Knockdown of SNHG1 improved DDP sensitivity in breast cells

**Figure 3.**
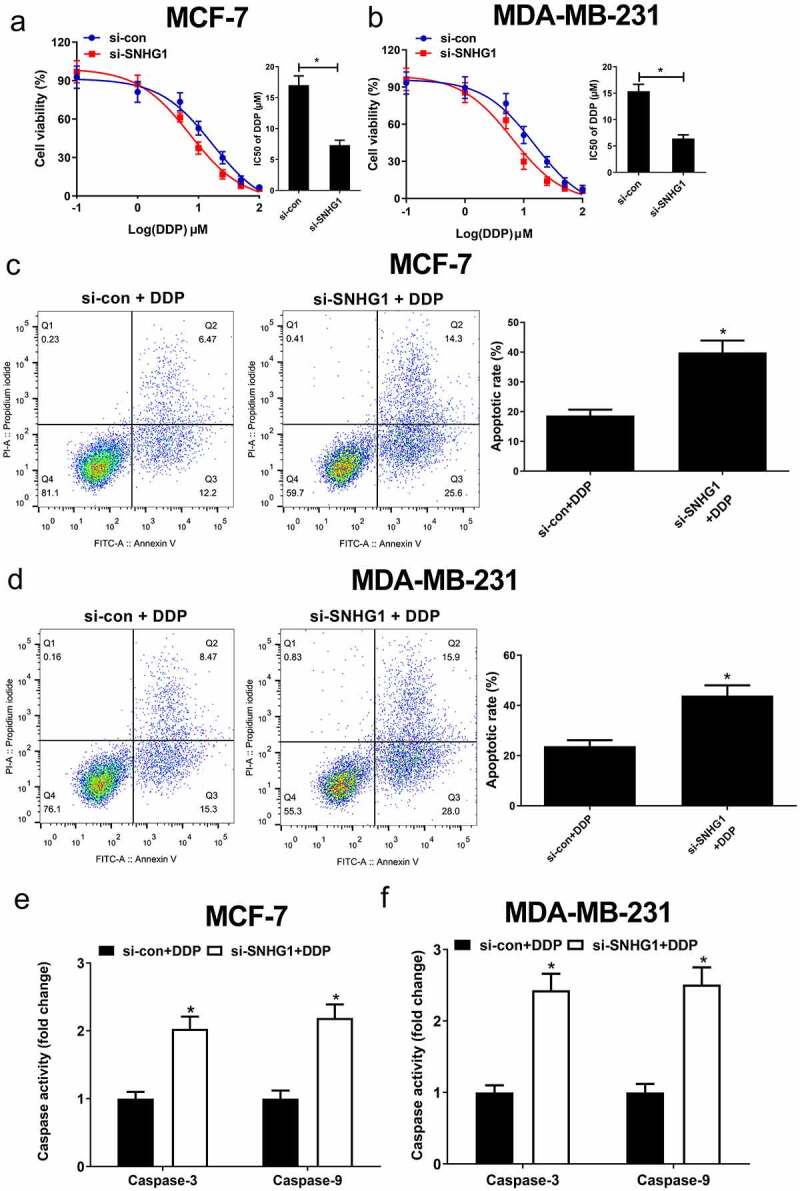
Knockdown of SNHG1 enhanced DDP sensitivity of breast cancer cells. (a and b) MCF-7 and MDA-MB-231 cells transfected with si-SNHG1 or si-con were treated with various concentrations of DDP (0.1, 1, 5, 10, 25, 50, 100 μM) for 48 h and cell viability was detected by MTT assay. Cell apoptosis was determined by flow cytometry analysis (c and d) and caspase activity assay (e and f) in si-SNHG1 or si-con transfected MCF-7 and MDA-MB-231 cells after treatment with 2 μM of DDP. *P < 0.05

To assess how SNHG1 influences DDP resistance in breast cancer cells, DDP sensitivity assays were performed in si-con or si-SNHG1 transfected breast cancer cells. The results showed that SNHG1 silencing noticeably strengthened breast cancer cell sensitivity to DDP (Figures 3(a,b)). Moreover, flow cytometry analysis found that SNHG1 inhibition strangely improved DDP-induced apoptosis (Figures 3(c,d)). Apoptosis-related proteins such as caspases are well known to be implicated with drug-induced apoptosis [26]. Our study found that SNHG1 depletion strikingly enhanced caspase-3/9 activity in breast cancer cells (Figures 3(e,f)). Totally, these findings indicated that SNHG1 downregulation improved breast cancer cell DDP sensitivity, highlighting its key regulatory roles in breast cancer chemoresistance.

### 3.4. SNHG1 epigenetically silenced miR-381

**Figure 4.**
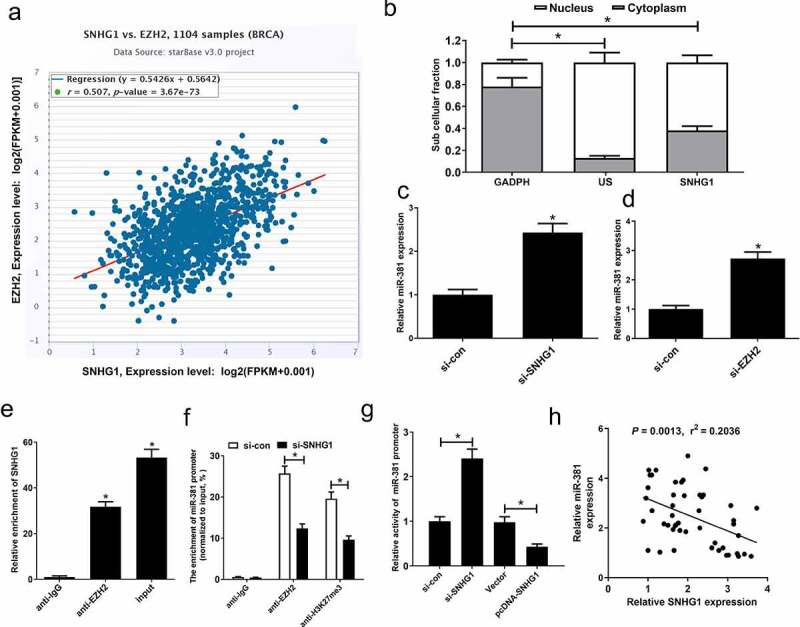
SNHG1 epigenetically silenced miR-381 in breast cancer cells. (a) Correlation analysis between SNHG1 and EZH2 in 1185 tumor tissue samples of breast cancer from TCGA datasets. (b) Fractionation of breast cancer cell lysates demonstrates the cytoplasm and nuclear expression of SNHG1. (c and d) miR-381 expression levels in MCF-7 cells transfected with (si-con or si-SNHG1) or (si-con or si-EZH2). RIP assays (e) were carried out to evaluate the interaction of SNHG1 and EZH2 in MCF-7 cells. (f) ChIP assay was performed to estimate the effect of SNHG1 on EZH2 occupancy and H3K27me3 enrichment on the promoter of miR-381 in MCF-7 cells. (g) Transcriptional activity of miR-381 promoter in MCF-7 cells upon SNHG1 knockdown or overexpression was detected by luciferase reporter assay. (h) Correlation analysis of SNHG1 and miR-381 expression in breast cancer tissues. *P < 0.05

It has been suggested that SNHG1 suppresses host cell gene expression through its ability to target the polycomb repressive complex 2 (PRC2) subunit EZH2 [20,21]. Chipbase analyses suggested that EZH2 was capable of binding miR-381, which could attribute to DNA hypermethylation [22]. Hence, we further investigated whether SNHG1 was an epigenetic suppressor of miR-381 via recruiting EZH2 in breast cancer cells. Firstly, Chipbase database analysis exhibited a close association between SNHG1 and EZH2 levels in breast cancer tissues (Figure 4(a)). Subcellular fractionation assay showed that SNHG1 possessed both a nuclear and cytoplasmic distribution in MCF-7 cells (Figure 4(b)). SNHG1 silencing or downregulation of EZH2 by si-EZH2 could strikingly increase the expression of miR-381 (Figure 4(c,d)). We additionally assessed the binding of SNHG1 and miR-381 using RIP assays. Results of RIP assay confirmed that SNHG1 could bind to EZH2 (Figure 4(e)). Furthermore, EZH2 can bind the promoter region of miR-381, resulting in H3K27me3 modification. However, knockdown of SNHG1 weakened EZH2 binding and H3K27me3 enrichment on the miR-381 promoter (Figure 4(f)). We next performed a luciferase reporter assay in MCF-7 cells, showing that SNHG1 knockdown increased miR-381 promoter activity, while the overexpression of SNHG1 inhibited miR-381 promoter activity (Figure 4(g)). SNHG1 and miR-381 expression were also negatively associated in breast cancer specimens (Figure 4(h)). These findings demonstrated that SNHG1 epigenetically suppressed miR-381 expression in breast cancer, suggesting that this axis may be one of the key regulatory processes governing oncogenic progression in breast cancer.

### 3.5. MiR-381 overexpression improved CDDP sensitivity and inhibited proliferative, migratory, and invasive activity

**Figure 5.**
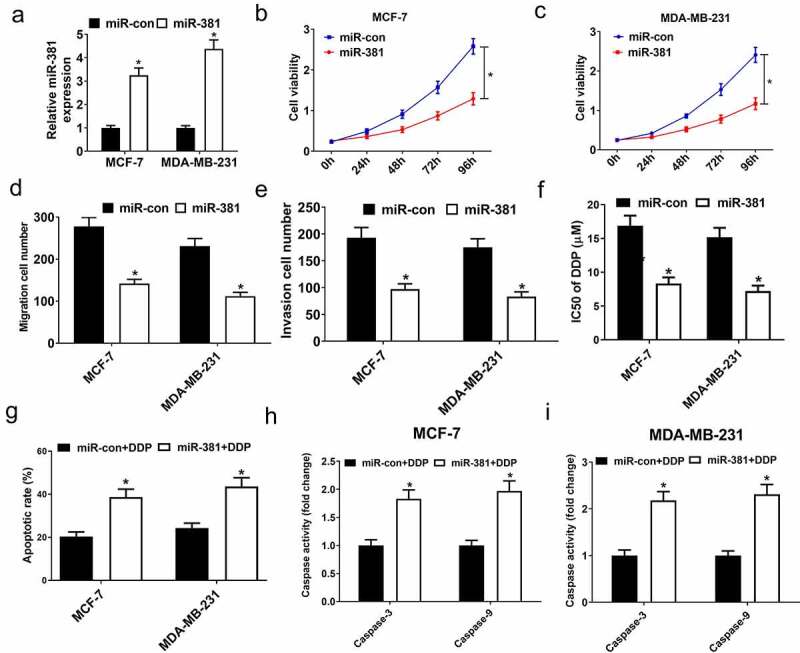
Overexpression of miR-381 suppressed proliferation, migration and invasion, and improved DDP sensitivity of breast cancer cells. MCF-7 and MDA-MB-231 cells were transfected with miR-con or miR-381, followed by determination of miR-381 expression by qRT-PCR analysis (a), cell viability by MTT assay (b and c), migration and invasion by Transwell migration and invasion assays (d and e), IC50 value by DDP sensitivity assay (f), and cell apoptosis by flow cytometry analysis (g) and caspase activity assay (h and i). *P < 0.05

To further define the role of miR-381 in breast cancer cells, cells were subject to exogenous miR-381 transfection which, as expected, increased miR-381 levels (Figure 5(a)). Additionally, the exogenous expression of miR-381 reduced the proliferative capacity of cells (Figure 5(b-c)), inhibited migration/invasion (Figure 5(d-e)), and enhanced DDP sensitivity (Figure 5(f)). Additionally, miR-381 overexpression enhanced DDP-induced apoptosis (Figure 5(g)) and increased caspase-3/9 activity (Figure 5(h-i)) in breast cancer cells. Together, elevated miR-381 expression suppressed tumor progression and enhanced breast cancer DDP sensitivity, emphasizing the value of miR-381 in breast cancer tumor progression and DDP resistance.

### 3.6. SNHG1 knockdown suppressed tumor progression and improved breast cancer DDP sensitivity via upregulating miR-381

**Figure 6.**
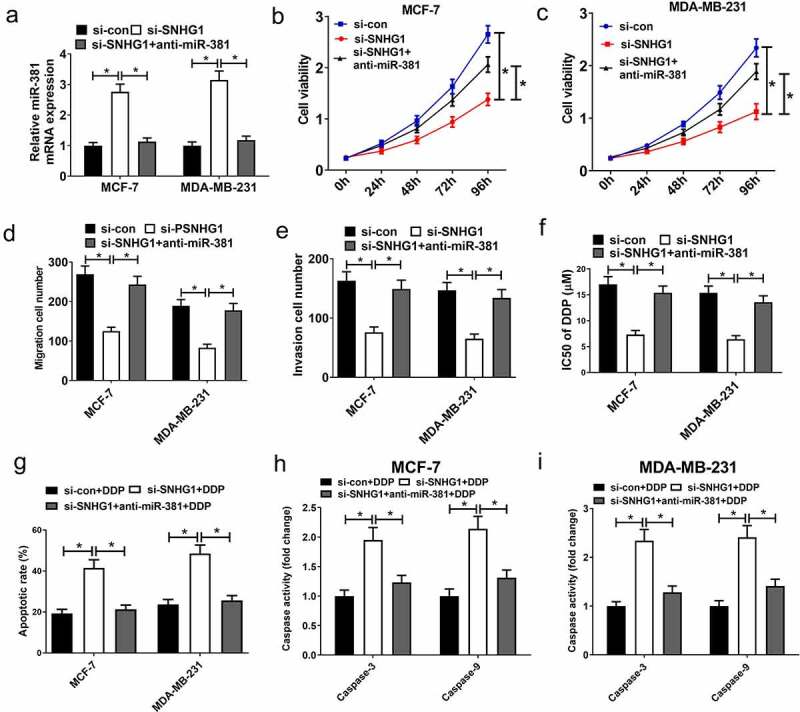
miR-381 knockdown reversed the effect of SNHG1 silencing on tumor progression and DDP sensitivity of breast cancer cells. MCF-7 and MDA-MB-231 cells were transfected with si-con, si-SNHG1 or si-SNHG1+ anti-miR-381, followed by determination of miR-381 expression by qRT-PCR analysis (a), cell viability by MTT assay (b and c), migration and invasion by Transwell migration and invasion assays (d and e), IC50 value by DDP sensitivity assay (f), and cell apoptosis by flow cytometry analysis (g) and caspase activity assay (h and i). *P < 0.05

**Figure 7.**
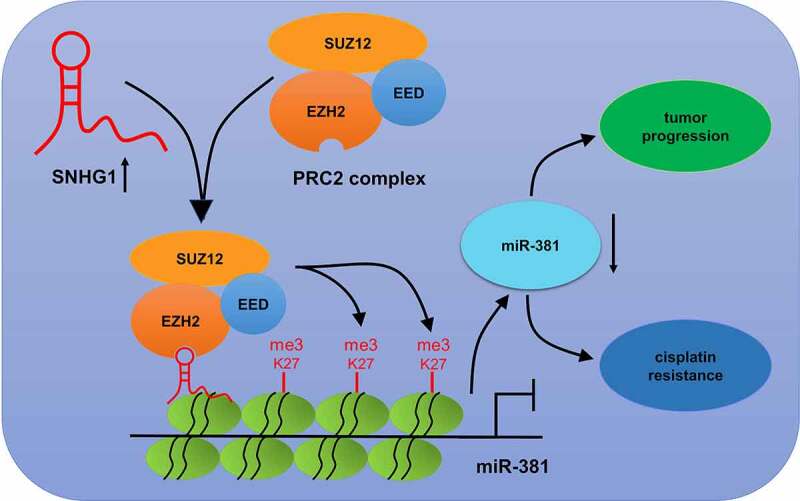
Schematic diagram of the mechanism of SNHG1 epigenetically silencing miR-381 in breast cancer

To verify if the functional impact of SNHG1 silencing in breast cancer is mediated by miR-381, cells were transfected with si-SNHG1 alone or together with anti-miR-381. Silencing of SNHG1 substantially increased miR-381 expression, while the opposite occurred upon anti-miR-381 transfection (Figure 6(a)). Downregulation of SNHG1 led to a loss of cell growth, migration and invasion. Importantly, the suppressive effect of SNHG1 inhibition was strikingly eliminated by miR-381 downregulation (Figure 6(b,e)). Moreover, miR-381 inhibition could reverse SNHG1 silencing-mediated enhancement of DDP sensitivity (Figure 6(f)). Furthermore, anti-miR-381 transfection extremely suppressed the silenced SNHG1-mediated enhancement of apoptosis (Figure 6(g)) and caspase-3/9 activity (Figure 6(h-i)). Collectively, these findings demonstrated that silencing SNHG1 reduced the metastatic and DDP-resistant phenotypes of breast cancer cells via upregulating miR-381 expression. Moreover, the schematic diagram of the mechanism through which SNHG1 promoted tumor progression and DDP resistance in breast cancer was shown in Figure 7.

## 4. Discussion

In breast cancer patients, the aggressive nature and chemoresistance have severely restricted the therapeutic outcome, as many patients are discovered when their disease is quite advanced and ineligible for curative treatment. Subsequently, the mechanism(s) of tumor progression and chemoresistance must be defined to improve therapeutics in the clinic. Herein, we show that SNHG1 was high-expressed in breast cancer tissues/cells, the silencing of which could prevent the metastatic and DDP-resistant phenotypes of breast cancer cells. Prominently, SNHG1 silencing suppressed tumor progression and sensitized breast cancer cells to DDP via epigenetically silencing miR-381. Thus, SNHG1 can function as an oncogenic gene in breast cancer and SNHG1 silencing may be effective for breast cancer chemotherapy.

The significance of lncRNAs as master regulators in carcinogenesis has been widely discussed, and its dysregulation has been considered as an important mechanism for cancer tumorigenesis [27]. Although lncRNAs are known to be linked to tumorigenesis, the mechanistic characterization of these RNAs has been limited and it is vital that they are more fully characterized in order to appropriately elucidate their cancer-causing potential. SNHG1 was reported to function as a cancer driver in a range of important human malignancies [28], including breast cancer [14,29]. Consistent with this, the results in our study suggested silencing of SNHG1 inhibited proliferative, invasive, and migratory activity. Nevertheless, our study first demonstrated that SNHG1 knockdown could overcome breast cancer DDP resistance, which may provide a novel therapeutic target to improve the outcome of breast cancer patients with DDP resistance. In addition, SNHG1 downregulation inhibited breast tumor growth *in vivo*. The culmination of these data suggests that SNHG1 represents a potential therapeutic target for breast cancer chemotherapy.

The mechanisms through which SNHG1 upregulation contributes to breast cancer progression and DDP resistance remains indescribable. Thus, we investigated the mechanistic roles of SNHG1. Recently, accumulating evidence shows that lncRNAs can epigenetically inhibit the expression of target genes via recruiting EZH2, ultimately affecting cell functions [30–32]. Prominently, SNHG1 could epigenetically suppress gene expression through recruiting EZH2 in various cancers [20,21]. The EZH2 histone methyltransferase serves as an epigenetic suppressor of gene expression via driving H3K27me3 enrichment in promoter regions [33,34]. Besides, miR-381 could attribute to DNA hypermethylation in cancers [18]. Moreover, EZH2 could bind and epigenetically silenced miR-381 in breast cancer [35]. However, the ability of SNHG1 to suppress miR-381 in breast cancer was not defined. We determined that SNHG1 or EZH2 knockdown could up-regulate miR-381 expression. RIP assay additionally established the ability of SNHG1 to interact with EZH2. Additionally, SNHG1 silencing enhanced miR-381 promoter activity via decreasing the recruiting of EZH2 and H3K27me3. These results suggested that SNHG1 epigenetically silences miR-381 through its ability to directly bind to and target EZH2 in breast cancer cells, demonstrating a novel epigenetic mechanism through which SNHG1 suppressed miR-381 expression at the transcriptional level via recruiting EZH2 and enriching H3K27me3 on the promotor region of miR-381. Growing evidence implicates miR-381 as a tumor suppressor [18,36–38]. Likewise, low-expressed miR-381 was implicated with tumor progression and DDP resistance in breast cancer [39–41]. Consistently, our work revealed that miR-381 reduced tumor progression, and enhanced the sensitivity of DDP. Furthermore, miR-381 inhibition alleviated the effects of SNHG1 silencing in breast cancer cells. This highlights that SNHG1 can suppress tumor progression and improve DDP sensitivity via the epigenetic silencing of miR-381 in breast cancer.

## 5. Conclusion

In this study, we show that SNHG1 contributed to tumor progression and DDP resistance in breast cancer cells by inhibiting miR-381 expression through a novel epigenetic mechanism, highlighting an attractive therapeutic target to obstruct tumor progression and overcome breast cancer DDP resistance.

## Highlights

(1) Knockdown of SNHG1 suppressed breast cancer tumor progression.

(2) SNHG1 contributed to DDP resistance in breast cancer cells.

(3) SNHG1 exerted its oncogene function through epigenetically silencing miR-381.

(4) Knockdown of SNHG1 inhibited growth of xenograft derived from breast cancer *in vivo*.

## References

[1] Siegel RL, Miller KD, PhD AJD. Cancer statistics, 2016. Ca A Cancer J Clinicians. 2016;66(1):7-30. 10.3322/caac.21332 26742998

[2] Sutter SA, Slinker A, Balumuka DD, et al. Surgical management of breast cancer in africa: a continent-wide review of intervention practices, barriers to care, and adjuvant therapy. J Glob Oncol. 2017;3(2):162–168. 28717754 10.1200/JGO.2016.003095 PMC5493273

[3] Szakács G, Paterson JK, Ludwig JA, et al. Targeting multidrug resistance in cancer. Nat Rev Drug Discov. 2006;5:219–234. 16518375 10.1038/nrd1984

[4] Coley HM. Mechanisms and strategies to overcome chemotherapy resistance in metastatic breast cancer. Cancer Treat Rev. 2008;34(4):378. 18367336 10.1016/j.ctrv.2008.01.007

[5] Schmitt AM, Chang HY. Long noncoding RNAs in cancer pathways. Cancer Cell. 2016;29(4):452–463. 27070700 10.1016/j.ccell.2016.03.010 PMC4831138

[6] Lin C, Yang L. Long noncoding RNA in cancer: wiring signaling circuitry. Trends Cell Biol. 2018;28(4):287–301. 29274663 10.1016/j.tcb.2017.11.008 PMC5869122

[7] Bartonicek N, Maag JL, Dinger ME. Long noncoding RNAs in cancer: mechanisms of action and technological advancements. Mol Cancer. 2016;15(1):43. 27233618 10.1186/s12943-016-0530-6 PMC4884374

[8] Sun Q, Hao Q, Prasanth KV. Nuclear long noncoding RNAs: key regulators of gene expression. Trends Genet. 2018;34(2):142–157. 29249332 10.1016/j.tig.2017.11.005 PMC6002860

[9] Wang H, Guan Z, He K, et al. LncRNA UCA1 in anti-cancer drug resistance. Oncotarget. 2017;8(38):64638–64650. 28969100 10.18632/oncotarget.18344 PMC5610032

[10] Le Q, Jin D, Cheng C, et al. Exosome-transmitted lncARSR promotes sunitinib resistance in renal cancer by acting as a competing endogenous RNA. Cancer Cell. 2016;29(5):653–668. 27117758 10.1016/j.ccell.2016.03.004

[11] Zhang Y, Song X, Wang X, et al. Silencing of LncRNA HULC enhances chemotherapy induced apoptosis in human gastric cancer. J Med Biochem. 2016;35(2):137–143. 28356873 10.1515/jomb-2015-0016 PMC5346790

[12] Jianping L, Zhipeng Z, Li X, et al. SNHG1 lncRNA negatively regulates miR-199a-3p to enhance CDK7 expression and promote cell proliferation in prostate cancer. Biochem Biophys Res Commun. 2017;487(1):146-152. 10.1016/j.bbrc.2017.03.169 28400279

[13] Zhang M, Wang W, Li T, et al. Long noncoding RNA SNHG1 predicts a poor prognosis and promotes hepatocellular carcinoma tumorigenesis. Biomed Pharmacother. 2016;80:73–79. 27133041 10.1016/j.biopha.2016.02.036

[14] Zheng S, Li M, Miao K, et al. SNHG1 contributes to proliferation and invasion by regulating miR-382 in breast cancer. Cancer Manag Res. 2019;11:5589. 31354360 10.2147/CMAR.S198624 PMC6590395

[15] Thomson DW, Dinger ME. Endogenous microRNA sponges: evidence and controversy. Nat Rev Genet. 2016;17(5):272–283. 27040487 10.1038/nrg.2016.20

[16] Kawamata T, Tomari Y. Making RISC. Trends Biochem Sci. 2010;35(7):368–376. 20395147 10.1016/j.tibs.2010.03.009

[17] Di Leva G, Garofalo M, Croce CM. MicroRNAs in cancer. Annu Rev Pathol. 2014;9(1):287–314. 24079833 10.1146/annurev-pathol-012513-104715 PMC4009396

[18] Xue Y, Xu W, Zhao W, et al. miR-381 inhibited breast cancer cells proliferation, epithelial-to-mesenchymal transition and metastasis by targeting CXCR4. Biomed Pharmacother. 2017;86:426–433. 28012397 10.1016/j.biopha.2016.12.051

[19] Yiwei L, Chen L, Feng M, et al. NEK2 promotes proliferation, migration and tumor growth of gastric cancer cells via regulating KDM5B/H3K4me3. Am J Cancer Res. 2019;9:2364–2378. 31815040 PMC6895449

[20] Li Z, Guo X, Wu S. Epigenetic silencing of KLF2 by long non-coding RNA SNHG1 inhibits periodontal ligament stem cell osteogenesis differentiation. Stem Cell Res Ther. 2020;11(1):435. 33028420 10.1186/s13287-020-01953-8 PMC7539403

[21] Li B, Li A, You Z, et al. Epigenetic silencing of CDKN1A and CDKN2B by SNHG1 promotes the cell cycle, migration and epithelial-mesenchymal transition progression of hepatocellular carcinoma. Cell Death Dis.2020;11(10):823. 10.1038/s41419-020-03031-6 PMC7532449 33009370

[22] Li J, Ying Y, Xie H, et al. Dual regulatory role of CCNA2 in modulating CDK6 and MET-mediated cell-cycle pathway and EMT progression is blocked by miR-381-3p in bladder cancer. FASEB J. 2018;33(1):1374–1388. 30138038 10.1096/fj.201800667R

[23] Shang A, Zhou C, Bian G, et al. miR-381-3p restrains cervical cancer progression by downregulating FGF7. J Cell Biochem. 2019;120(1):778–789. 30161290 10.1002/jcb.27438

[24] Wang WJ, Yao Y, Jiang LL, et al. Knockdown of lymphoid enhancer factor 1 inhibits colon cancer progression in vitro and in vivo. PLoS One. 2013;8(10):e76596. 24098538 10.1371/journal.pone.0076596 PMC3788715

[25] Xu M, Chen X, Lin K, et al. The long noncoding RNA SNHG1 regulates colorectal cancer cell growth through interactions with EZH2 and miR-154-5p. Mol Cancer. 2018;17(1):141. 30266084 10.1186/s12943-018-0894-x PMC6162892

[26] Los M, Herr I, Friesen C, et al. Cross-resistance of CD95- and drug-induced apoptosis as a consequence of deficient activation of caspases (ICE/Ced-3 proteases). Blood. 1997;90(8):3118. 9376593

[27] Lorenzi L, Avila Cobos F, Decock A, et al. Long noncoding RNA expression profiling in cancer: challenges and opportunities. Genes Chromosomes Cancer. 2019;58(4):191–199. 30461116 10.1002/gcc.22709

[28] Dong B, Chen X, Zhang Y, et al. The prognostic value of lncRNA SNHG1 in cancer patients: a meta-analysis. BMC Cancer. 2019;19(1):780. 31391030 10.1186/s12885-019-5987-4 PMC6686246

[29] Xiong X, Feng Y, Li L, et al. Long non‑coding RNA SNHG1 promotes breast cancer progression by regulation of LMO4. Oncol Rep. 2020;43:1503–1515. 32323846 10.3892/or.2020.7530 PMC7107776

[30] Xu Y, Yao Y, Jiang X, et al. SP1-induced upregulation of lncRNA SPRY4-IT1 exerts oncogenic properties by scaffolding EZH2/LSD1/DNMT1 and sponging miR-101-3p in cholangiocarcinoma. J Exp Clin Cancer Res. 2018;37(1):81. 29642935 10.1186/s13046-018-0747-x PMC5896100

[31] Zheng W, Yu A. EZH2-mediated suppression of lncRNA-LET promotes cell apoptosis and inhibits the proliferation of post-burn skin fibroblasts. Int J Mol Med. 2018;41:1949–1957. 29393360 10.3892/ijmm.2018.3425 PMC5810232

[32] Luo J, Xiang H. LncRNA MYLK-AS1 acts as an oncogene by epigenetically silencing large tumor suppressor 2 (LATS2) in gastric cancer. Bioengineered. 2021;12(1):3101–3112. 34181498 10.1080/21655979.2021.1944019 PMC8806516

[33] Cao R, Wang L, Wang H, et al. Role of histone H3 lysine 27 methylation in polycomb-group silencing. Science. 2002;300:131. 10.1126/science.1076997 12351676

[34] Cao R, Zhang Y. The functions of E(Z)/EZH2-mediated methylation of lysine 27 in histone H3. Curr Opin Genet Dev. 2004;14(2):155–164. 15196462 10.1016/j.gde.2004.02.001

[35] Dou D, Ge X, Wang X, et al. EZH2 contributes to cisplatin resistance in breast cancer by epigenetically suppressing miR-381 expression. Onco Targets Ther. 2019;14:9627–9637. 10.2147/OTT.S214104 PMC6859472 32009798

[36] Tian C, Li J, Ren L, et al. MicroRNA-381 serves as a prognostic factor and inhibits migration and invasion in non-small cell lung cancer by targeting LRH-1. Oncol Rep. 2017;38:3071–3077. 29048619 10.3892/or.2017.5956

[37] Cao Q, Liu F, Ji K, et al. MicroRNA-381 inhibits the metastasis of gastric cancer by targeting TMEM16A expression. J Exp Clin Cancer Res. 2017;36(1):29. 28193228 10.1186/s13046-017-0499-z PMC5307754

[38] He X, Wei Y, Wang Y, et al. MiR-381 functions as a tumor suppressor in colorectal cancer by targeting Twist1. Onco Targets Ther. 2016;9:1231. 27094913 10.2147/OTT.S99228 PMC4789845

[39] Wu M, Fan B, Guo Q, et al. Knockdown of SETDB1 inhibits breast cancer progression by miR-381-3p-related regulation. Biol Res. 2018;51(1):39. 30309377 10.1186/s40659-018-0189-0 PMC6180515

[40] Xue Y, Xu W, Zhao W, et al. miR-381 inhibited breast cancer cells proliferation, epithelial-to-mesenchymal transition and metastasis by targeting CXCR4. Biomed Pharmacothe. 2017;86:426–433. 10.1016/j.biopha.2016.12.051 28012397

[41] Sarrafzadeh S, Geranpayeh L, Ghafouri-Fard S. Expression analysis of long non-coding PCAT-1in breast cancer. Int J Hematol Oncol Stem Cell Res. 2017;11:185. 28989584 PMC5625468

